# How should a principal reward and support agents when firm performance is characterized by success or failure?

**DOI:** 10.1002/mde.3006

**Published:** 2019-02-22

**Authors:** Christian Schmid, Yigal Gerchak

**Affiliations:** ^1^ Department of Strategy and Innovation WU Vienna University of Economics and Business Vienna Austria; ^2^ Department of Industrial Engineering Tel‐Aviv University Tel‐Aviv Israel

## Abstract

Principal‐agent models with multiple agents typically assume that the principal wishes to maximize the sum of the agents' achievements (net of the rewards paid to them). But in many settings, like R&D, all that the principal “needs” is that at least one agent will be “successful.” We identify settings where the principal actually wants agents to refrain from exerting high effort in order to save expected compensation. We show that the number of agents can decrease in the project's value for the principal. We also consider sequential efforts and investigate settings where the principal can provide support to agents.

## 1 INTRODUCTION

In many human undertakings, the only result that really matters is whether the activity ended in success or failure; the definition of which is often quite clear. In such environments, the cardinal value of the achievement, beyond constituting a success or not, is immaterial. Sometimes, success is the achievement of some predetermined threshold. Examples include many types of R&D undertakings, which attempt to develop a device or procedure that will achieve a certain performance level (see, e.g., Abernathy & Rosenbloom, 1969; Gerchak & Kilgour, 1999, 2014). In such settings, the task is completed as soon as at least one of multiple attempts is successful. The objective of achieving success is related to, but distinct from, settings where only the highest achievement of multiple parties matters (Nelson, 1961; Dasgupta & Maskin, 1987; Bard, 1985; Terwiesch & Xu, 2008).

In agency settings where a principal deploys several “parallel” agents, she may also only be interested whether any of them will succeed (referred to as “OR” in the computer science literature, e.g., Babaioff, Feldman, Nisan, & Winter, 2012) as opposed to a principal wanting the number of successful agents to be as large as possible, in line with the common “sum of outputs” objective. Many organizations make use of several agents in parallel (e.g., R&D teams and athletes attempting to achieve certain thresholds). These agents sometimes cannot cooperate due to geographic and/or cultural barriers and competition within the organization (and they also do not report partial results to others). Of multiple parallel R&D teams that a firm (or country) engages, it may only matter whether at least one of them achieves the goal. For instance, Gerchak and Kilgour (1999) provide real‐world examples of firms employing independent parallel R&D teams. That is also the motivation for our model. The endeavor is successful if at least one of the agents succeeds. However, the principal will have to reward all successful agents.

Our agents do not compete with each other, as the reward depends on their individual achievement. Thus, our setting is not a contest (Glazer & Hassin, 1988; Lazear & Rosen, 1981; Canbolat, Golany, Mund, & Rothblum, 2012; Moldovanu & Sela, 2001). The occasional practice of firms employing more than one auditor to examine its entire books instead of dividing their books among auditors (“dual audits”) is also an example of a parallel strategy where it is sufficient that at least one auditor detects relevant irregularities.

Several articles have investigated moral hazard situations (where the principal cannot observe the agents' effort) in multiagent settings to enhance our understanding of collective effort (e.g., Che & Yoo, 2001; Baldenius, Glover, & Xue, 2016) or relative performance evaluation (e.g., Bartling, 2012; Glover, 2012). Sometimes, it is assumed that agents work “together” to achieve an output. For instance, Itoh (1991) provides arguments for teamwork. He shows that it can be optimal for a principal to incentivize agents to help other agents in accomplishing their tasks. In contrast to settings with collective effort as in Che and Yoo (2001) and Baldenius et al. (2016), we consider agents who are hired to work independently.

Gerchak and Schmid (2016) consider a principal who is only interested in the highest (or lowest ‐ “AND”) achievement of any agent but on a continuous scale. Here, it is assumed that there exists a threshold, such that only the probability of achieving that threshold matters to the principal.

Typically, multiagent models with binary effort levels *assume* that a principal always wants all agents to provide high effort (e.g., Che & Yoo, 2001; Glover, 2012). We derive explicit conditions when the principal prefers agents to *refrain from exerting high effort* in order to save expected compensation. In that sense, we enlarge the outcome space for possible equilibria as compared with previous literature on multiagent settings.

Besides designing the incentive system, principals often decide how many parallel agents to engage in a specific task. More agents will increase administration costs of employment. However, this might not be the only downside of a greater workforce. Although employing more agents increases the probability of success, the number of potential rewards is increasing.

The work of Babaioff et al. (2012), in combinatorial economics, investigates a setting similar to ours with their nonobservable “OR” technology. Their focus is on how many (and which) agents, of an available group, should be contracted to exert high effort. In their “OR” setting, the number of agents that should be hired increases one by one as a principal's profit in case of success increases. We characterize in detail when two agents, and of what type, are superior/inferior to a single agent, as a function of the administration cost. We show that if hiring an agent induces additional administration costs, the number of agents does not necessarily grow “smoothly” with the project's value for the principal. For example, it might be favorable to switch from two low‐effort agents (e.g., part‐time workers) to one high‐effort worker (e.g., full‐time worker) as the project's value increases. A “follow‐up” article, Babaioff, Feldman, and Nisan (2010), deals with mixed strategies. They do not specifically consider a principal who can provide additional support to the agents.

Levitt (1995) examines a LEN framework where only the best outcome of multiple agents matters to the principal. He shows that it can be optimal to pay asymmetric wages for identical agents assigned to the same task and investigates under what conditions a principal prefers to employ a single agent rather than two symmetric agents. As Levitt's model does not provide a closed‐form solution of the optimal asymmetric incentive scheme, however, he has to limit the latter investigation to some special cases. Our simpler model (without common production shocks and with binary outcomes) allows for a general analysis of whether to hire one or two agents.

When success by one of multiple agents is sufficient for a principal, she might prefer to hire and incentivize independent agents in sequence. The principal then faces a simple trade‐off. If the first agent was successful, no other agent has to be hired and rewarded for possible (unnecessary) success subsequently. Yet success at a later point in time typically reduces the benefits for a firm. In Section 5, we extend our basic model by allowing engaging agents sequentially. We show that the discount factor for later revenues always critically affects the principal's decision whether to employ agents simultaneously or sequentially. The issue of parallel versus sequential hiring especially relates to parallel and sequential R&D strategies as discussed, for instance, by Abernathy and Rosenbloom (1968) and Loch, Terwiesch, and Thomke (2001). In our model, we abstract from learning effects that typically promote sequential R&D strategies. Alternatively, Bose, Pal, and Sappington (2010) show that sequential efforts are beneficial for the principal if the agents' contributions serve as complements in their joint performance. The first mover anticipates that high effort increases the second agent's productivity and thereby the contribution to the joint performance. Hence, the first mover experiences an additional motivation to work hard, which allows the principal to save compensation costs. In contrast, in our setting, all performance measures are individual and the agents' efforts always constitute substitutes. Hence, without learning effects, the benefit of sequential efforts arises by the real option of saving compensation because already one agent's success is sufficient for the principal.

Section 2 introduces our basic model of success and failure and explores the optimal reward structure when only one agent is employed, as a reference point to multiagent models. In Section 3, we investigate the multiagent environment with a given number of parallel agents. In Section 4, we investigate how many homogeneous agents the principal prefers to hire and identify conditions where the principal refrains from employing additional agents. In Section 5, we allow agents to deliver effort sequentially and explore when the real option of not hiring a second agent after prior success outweighs the expected reduction in the project's value due to discounting. Section 6 extends our analysis of a setting with a fixed number of agents by allowing for asymmetric agents that differ in their ability. In Sections 7, we extend our analysis by allowing the principal to support agents. We focus on situations where the principal has the opportunity to “help” the agents, rather than on cooperation among agents. The support is assumed to have a similar effect when the agent exerts low effort to when he exerts high effort. The costs associated with support are increasing and convex, which seems realistic. In Section 7.1, the probability of success is assumed to be proportional to the level of support, possibly with asymmetric agents. Laux (2017) uses a similar concept of support but assumes that the effectiveness of support depends on the agents' (unequal) abilities.^1^ In Section 7.2, we assume decreasing returns to the principal's level of support. Section 8 concludes and suggests avenues for future research. The Appendix provides all proofs.

## 2 BASIC SET‐UP

Any agent (A) employed by a principal (P) makes an unobservable choice between two types of effort *a*  ∈  {*L*,*H*}. A's private costs of effort are *c*(*L*) and *c*(*H*), respectively, where high effort is more costly to A. For the most part of the analysis, we set *c*(*L*)  =  0 and *c*(*H*)  =  1; only in Section 6, we consider heterogeneous agents that differ in their costs of effort. The outcome of A's effort can either be success or failure. An agent choosing high (low) effort will succeed with probability *p*_*H*_ (*p*_*L*_), and with probability 1 − *p*_*H*_ (1 − *p*_*L*_), the agent will fail, where *p*_*H*_  >  *p*_*L*_. In case of success, A is rewarded by P with a prize *w*_*S*_  ≥  0, whereas he receives *w*_*F*_  ≥  0 in case of failure. When A fails, he cannot be fined and made liable. One can view *w*_*F*_ as a “basic” wage and (*w*_*S*_ − *w*_*F*_) as a bonus for success. We assume that *w*_*F*_ and *w*_*S*_ correspond to the agents' respective utilities of the prizes. When the agents' preferences are represented by von Neumann‐Morgenstern utilities, these utilities are invariant with respect to linear transformations. Thus, one can assign arbitrary utilities to two prize values, and thus, risk attitude does not play a role here. Although P cannot observe the agents' effort choice, all parameters are common knowledge.

Let *D*_*S*_  >  0 denote P's value from the project in case of at least one agent's success, whereas P receives zero if all agents fail. If multiple agents participate in the project and some succeed, P's value remains *D*_*S*_ regardless of whether one or several agents were successful. Still, P has to compensate every agent who succeeds.

Additionally, P incurs administration costs of *d*  >  0, which comprise recruitment and training costs, for every agent employed, independent of the agent's effort. As A's personal cost of providing low effort is zero, this assumption guarantees that P does not want to employ infinitely many low‐effort workers. In practice, administration costs are commonly observed and comprise recruitment costs, nonwage labor costs, lump‐sum taxes, and basic working equipment, or office space. The administration costs can be ignored whenever we investigate the reward structure of a fixed number of agents, as the administration costs are sunk at that point. Only if P has to decide on the optimal workforce size must she incorporate *d* into her decision making.

### 2.1 Single agent

First, we will investigate the reward structure in a basic model with a single agent. The participation constraints (PCs) for an agent supplying high or low effort are, 

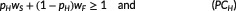


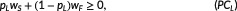
 respectively. The (ex ante) PC guarantees that, in expectation, the agent receives at least his reservation utility, which is equal to zero.

As the agent's costs of low effort are zero, an agent providing low effort does not have to be compensated. Thus, P will set *w*_*S*_  =  *w*_*F*_  =  0 and P's expected profit is *p*_*L*_ *D*_*S*_ − *d*. In order for A to exert high effort, (*PC* _*H*_) must hold and A must be better off by exerting high effort; thus, P faces an additional incentive compatibility constraint: 

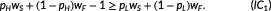



Because prizes have to be nonnegative, the (*IC* _1_) constraint guarantees that (*PC* _*H*_) holds. P's optimization problem is then to minimize expected rewards paid to the agent. P will optimally set *w*_*F*_  =  0 and the prize for success as small as possible, that is, *w*_*S*_  =  1/(*p*_*H*_ − *p*_*L*_)  =  :*W*. P's expected profit in this equilibrium is then $p_{H}\left(D_{S}-W\right)-d$. Comparing the equilibrium outcomes, we see that P will prefer the agent to exert high effort if and only if

(1) $$D_{S}>\frac{p_{H}}{{\left(p_{H}-p_{L}\right)}^{2}}:=D_{S}(1).$$

## 3 MULTIPLE HOMOGENEOUS AGENTS

Next, we consider the basic model with a fixed number of multiple symmetric agents who choose their effort level simultaneously. We do not restrict our analysis to symmetric contracts, so agents can be rewarded differently. If two agents can be treated differently, in our model, P offers Agent 1 rewards $w_{S}^{1}$ and $w_{F}^{1}$, whereas Agent 2 receives $w_{S}^{2}$ and $w_{F}^{2}$. Missing superscripts indicate that agents receive the same contracts.

As P observes the agents' individual success, the reward for each agent could depend on both agents' outcomes and thus their peers' performances. However, in absence of a common shock, P cannot benefit from relative performance evaluation (see Glover, 2012). Consequently, our setting allows us to restrict attention to rewards $w_{S}^{i}$ and $w_{F}^{i}$, *i*  ∈  {1,2}, which reflect individual performance evaluation.

Any agent *i* who is successful will be rewarded with $w_{S}^{i}$, and with $w_{F}^{i}$ if the agent fails. The other agents' effort and success do not influence agent *i*'s optimization problem and the reward structure will depend on whether P wants agents to exert high effort or not. If P prefers agents to exert high effort, (*IC* _1_) must hold and thus the prizes will be $w_{F}^{i}=0$ and $w_{S}^{i}=W$. If, in contrast, agents should provide low effort, P only has to make sure that the participation constraint for low effort will hold. Consequently, P will optimally set $w_{S}^{i}=w_{F}^{i}=0$.

Suppose first that P has to write symmetric contracts for all agents. Then, when two agents participate, comparing P's expected profits, we find that high effort is desirable if and only if

(2) $$\pi (H,H)>\pi (L,L)\;\Leftrightarrow \;D_{S}>D_{S}(2):=\frac{2p_{H}}{{\left(p_{H}-p_{L}\right)}^{2}\left(2-p_{H}-p_{L}\right)},$$

where *π*(*a*_1_,*a*_2_) denotes P's expected profit when Agent 1 (2) provides effort *a*_1_ (*a*_2_) with (*a*_1_,*a*_2_)  ∈  {*L*,*H*} × {*L*,*H*}, and *D*_*S*_(2)  =  *D*_*S*_(1)·2/(2 − *p*_*H*_ − *p*_*L*_)  >  *D*_*S*_(1). Both profits *π*(*H*,*H*)  =  *p*_*H*_(2 − *p*_*H*_)*D*_*S*_ − 2*p*_*H*_/(*p*_*H*_ − *p*_*L*_) − 2*d* and *π*(*L*,*L*)  =  *p*_*L*_(2 − *p*_*L*_)*D*_*S*_ − 2*d* show a linear increase in *D*_*S*_, but *π*(*H*,*H*) with a steeper slope. Also, *∂π*(*H*,*H*)/*∂p*_*H*_  =  2*D*_*S*_(1 − *p*_*H*_) + 2*p*_*L*_/(*p*_*H*_ − *p*_*L*_)^2^  >  0, whereas an increase in *p*_*H*_ does not change P's expected profit in case of two low‐effort agents.

Generally, P prefers *n* agents to exert high rather than low effort if and only if

(3) $$D_{S}>D_{S}(n):=\frac{np_{H}}{\left(p_{H}-p_{L}\right)\left({\left(1-p_{L}\right)}^{n}-{\left(1-p_{H}\right)}^{n}\right)}.$$

So, as in the single‐agent setting, only if the principal's value from the project is sufficiently high will she incentivize agents to provide high effort.

Lemma 1 The principal's value from the project required to prefer agents to exert high effort is increasing with the number of agents employed.

Lemma 1 indicates that when the number of agents employed at a project increases, the parameter region where P wants all agents to exert high effort becomes smaller.

Now, we allow that Agent 1 receives a different contract than Agent 2 (even though all agents are equally skilled). Without loss of generality, P wants Agent 1 to exert high effort, whereas Agent 2 should provide low effort. Employing a low‐effort (for example, part‐time) worker still causes administration costs of *d*. As a low‐effort worker does not have to be compensated, P will set $w_{S}^{2}=w_{F}^{2}=0$, whereas $w_{F}^{1}=0$ and $w_{S}^{1}=W$, to incentivize Agent 1 to provide high effort. Proposition 1 establishes that P will not always treat symmetric agents equally.

Proposition 1 There exists a nonempty region where the principal prefers asymmetric contracts for two symmetric agents, that is, π(H,L)>max{π(H,H),π(L,L)} for *D*_*S*_  ∈  (*A*_1_,*A*_2_) where *A*_1_ and *A*_2_ are values such that *A*_1_  <  *D*_*S*_(2)  <  *A*_2_. The Appendix provides the values for *A*_1_ and *A*_2_.

## 4 ONE VERSUS TWO AGENTS

In the previous section, we assumed that the number of agents employed is fixed. In practice, however, P is often not only responsible for designing the incentive systems but also decides how many agents are hired in the first place. The trade‐off is between the positive contribution of additional agents to the probability of at least one success, and the need to reward (possibly) more successful agents and pay additional administration costs. We will restrict our investigations to scenarios with two symmetric agents and compare it with employing a single agent. For simplicity, we will focus on equilibria in pure strategies but allow for asymmetric contracts. As hiring takes place before P sets prizes, we solve for the optimal employment decision by backward induction, where optimal incentives have already been established in the previous sections.

In the single agent case, we have already established that *π*(*H*)  >  *π*(*L*) if *D*_*S*_  >  *D*_*S*_(1)  =  *p*_*H*_/(*p*_*H*_ − *p*_*L*_)^2^. For two agents, we have already shown that *π*(*H*,*H*)  >  *π*(*L*,*L*) if *D*_*S*_  >  *D*_*S*_(2), where *D*_*S*_(2)  =  2*p*_*H*_/[(*p*_*H*_ − *p*_*L*_)^2^(2 − *p*_*H*_ − *p*_*L*_)], and similarly, *π*(*H*,*L*)  >  *π*(*L*,*L*) if *D*_*S*_  >  *A*_1_ and *π*(*H*,*H*)  >  *π*(*H*,*L*) if *D*_*S*_  >  *A*_2_, where *D*(1)  <  *A*_1_  <  *D*(2)  <  *A*_2_. However, we also have to compare when P prefers to hire a single agent to the scenarios of hiring two agents. P's preferred number of agents will depend on the administration costs *d*. To avoid numerous case distinctions, we will assume, in the sequel, that

(4) $$p_{H}>2p_{L}-p_{L}^{2}.$$

This condition assures that the probability of success of one high‐effort agent is larger than the joint probability of success of two low‐effort agents.^2^

While agents providing low effort do not have to be compensated, every agent causes administration cost of *d*. Thus, P prefers to employ two low‐effort agents instead of one if *d* is sufficiently small. Formally, *π*(*L*,*L*)  >  *π*(*L*) if $d<D_{S}(p_{L}-p_{L}^{2})$, or equivalently, $D_{S}>d/(p_{L}-p_{L}^{2})=:V_{1}$. Similarly, P prefers two high‐effort agents to one high‐effort agent, that is *π*(*H*,*H*)  >  *π*(*H*), if *D*_*S*_  >  (*p*_*H*_ − *d*(*p*_*H*_ − *p*_*L*_))/[(*p*_*H*_ − *p*_*L*_)*p*_*H*_(1 − *p*_*H*_)]  =  :*V*_5_. Similar calculations show that *π*(*L*,*L*)  >  *π*(*H*) if $D_{S}>(p_{H}-d(p_{H}-p_{L}))/[(p_{H}-p_{L})(p_{H}-2p_{L}+p_{L}^{2})]=:V_{2}$. Finally, *π*(*H*,*H*)  >  *π*(*L*) if $D_{S}>(d(p_{H}-p_{L})+2p_{H})/[(p_{H}-p_{L})(2p_{H}-p_{H}^{2}-p_{L})]$ (>*D*_*S*_(1)).

Comparing a single agent to asymmetric contracts for two agents shows that *π*(*H*,*L*)  >  *π*(*L*) if *D*_*S*_  >  (*p*_*H*_ + *d*(*p*_*H*_ − *p*_*L*_))/[(*p*_*H*_ − *p*_*L*_)*p*_*H*_(1 − *p*_*L*_)]  =  :*V*_3_, and *π*(*H*,*L*)  >  *π*(*H*) if *D*_*S*_  >  *d*/[(1 − *p*_*H*_)*p*_*L*_]  =  :*V*_4_.

Some calculations show that *A*_1_  =  *V*_2_ if *d*  =  *p*_*H*_(1 − *p*_*H*_)*p*_*L*_/[(*p*_*H*_ − *p*_*L*_)^2^(1 − *p*_*L*_)]  =  :*d*_1_, *D*(1)  =  *V*_2_ if *d*  =  *p*_*H*_(1 − *p*_*L*_)*p*_*L*_/(*p*_*H*_ − *p*_*L*_)^2^  =  :*d*_2_, and *A*_2_  =  *V*_5_ if *d*  =  *p*_*H*_ *p*_*L*_/(*p*_*H*_ − *p*_*L*_)^2^  =  :*d*_3_, where condition (4) guarantees that 0  <  *d*_1_  <  *d*_2_  <  *d*_3_.

In order for P to hire any agent, her expected profit has to be positive. In particular, P prefers hiring one low‐effort agent rather than not if *D*_*S*_  >  *d*/*p*_*L*_. Similarly, P prefers one high‐effort agent over not hiring this agent if *D*_*S*_  >  *W* + *d*/*p*_*H*_. Comparing these two thresholds for the project's value, we find that the latter is smaller if and only if *d*  >  *d*_3_.

Dependent on the level of the administration costs, P's optimal choice of the number of agents and the preferred level of effort(s) vary significantly. For instance, if *d* is sufficiently small, that is, if *d*  <  *d*_1_ holds, we find that *V*_1_ has the smallest value of all thresholds for possible regime changes.^3^ Thus, P prefers a single agent exerting low effort if *D*_*S*_  <  *V*_1_. Because P's profits are positive if *D*_*S*_  >  *d*/*p*_*L*_, she will hire a single agent providing low effort if *D*_*S*_  ∈  [*d*/*p*_*L*_,*V*_1_]. If *D*_*S*_ increases above *V*_1_, P will hire an additional agent, but both will provide low effort. Because for *d*  <  *d*_1_, *V*_2_  >  *A*_1_ holds, this strategy is dominant for *D*_*S*_  ∈  [*V*_1_,*A*_1_]. For *D*_*S*_  ∈  [*A*_1_,*A*_2_], P prefers different contracts if two agents are hired. As *D*(1)  <  *A*_1_ and *V*_4_  <  *A*_1_ hold in this scenario, P also prefers asymmetric contracts over hiring a single agent. Because *V*_3_,*V*_4_  <  *A*_2_, P will change to employing two high‐effort agents if *D*_*S*_ increases further such that *D*_*S*_  >  *A*_2_.

Proposition 2 describes P's preferred workforce and effort levels for all possible values of the administration costs.

Proposition 2 If $p_{H}>2p_{L}-p_{L}^{2}$ holds, P's choice between one versus two agents is as follows.

(i) If *d*  <  *d*_1_, (*L*) is optimal for *d*/*p*_*L*_  <  *D*_*S*_  <  *V*_1_, (*L*,*L*) for *D*_*S*_  ∈  [*V*_1_,*A*_1_], (*H*,*L*) for *D*_*S*_  ∈  [*A*_1_,*A*_2_], and (*H*,*H*) for *D*_*S*_  >  *A*_2_.
(ii) If *d*  ∈  [*d*_1_,*d*_2_], (*L*) is optimal for *d*/*p*_*L*_  <  *D*_*S*_  <  *V*_1_, (*L*,*L*) for *D*_*S*_  ∈  [*V*_1_,*V*_2_], (*H*) for *D*_*S*_  ∈  [*V*_2_,*V*_4_], (*H*,*L*) for *D*_*S*_  ∈  [*V*_4_,*A*_2_], and (*H*,*H*) for *D*_*S*_  >  *A*_2_.
(iii) If *d*  ∈  [*d*_2_,*d*_3_], (*L*) is optimal for *d*/*p*_*L*_  <  *D*_*S*_  <  *D*_*S*_(1), (*H*) for *D*_*S*_  ∈  [*D*_*S*_(1),*V*_4_], (*H*,*L*) for *D*_*S*_  ∈  [*V*_4_,*A*_2_], and (*H*,*H*) for *D*_*S*_  >  *A*_2_.
(iv) If *d*  >  *d*_3_, (*H*) is optimal for *D*_*S*_  ∈  [*W* + *d*/*p*_*H*_,*V*_5_], and (*H*,*H*) for *D*_*S*_  >  *V*_5_.

We see that, dependent on the administration costs, P's preferred workforce and types of effort vary significantly. For instance, for *d*  ∈  [*d*_1_,*d*_2_], as *D*_*S*_ increases, P will change from hiring one low‐effort agent to two low‐effort agents at *V*_1_, to a single high‐effort agent at the threshold *V*_2_, to two differently rewarded agents at *V*_4_ and two high‐effort agents at *A*_2_. Hence, Proposition 2 establishes that P's hiring decision does not necessarily follow a monotone pattern. Rather, it is possible that the number of agents decreases as the project's value increases.

**Figure 1:**
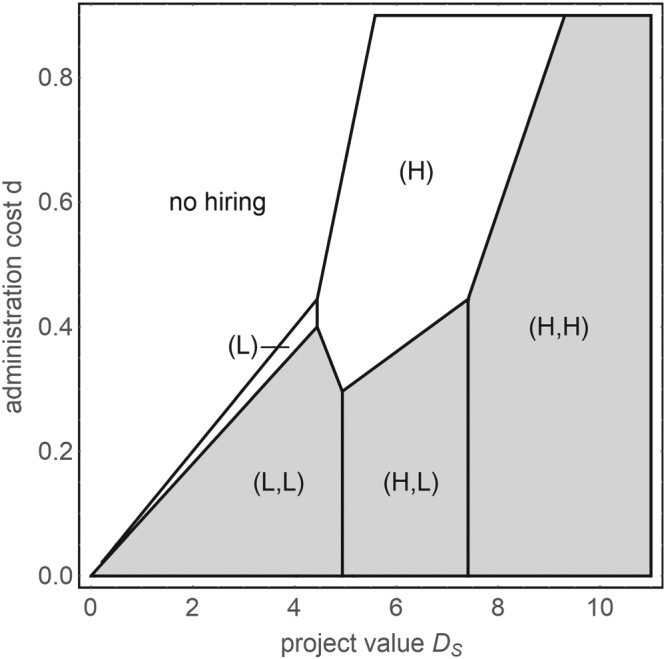
Employment of one versus two agents for varying magnitudes of the project's value D_S_ and administration costs d. The parameters are p_H_  =  4/10 and p_L_  =  1/10

For the specific probabilities of success *p*_*H*_  =  0.4 and *p*_*L*_  =  0.1, we have *d*_1_  =  8/27≈0.30, *d*_2_  =  0.40, *d*_3_  =  4/9≈0.44, *A*_1_  =  400/81≈4.9, *A*_2_  =  200/27≈7.4, *V*_1_  =  100*d*/9≈11.1*d*, *V*_2_  =  (400 − 300*d*)/63, *V*_3_  =  (100 + 75*d*)/27, *V*_4_  =  50*d*/3≈16.7*d* and *V*_5_  =  (100 − 75*d*)/18. P's expected profit *π*(*L*) is positive for *D*_*S*_  >  0.4*d*, and *π*(*H*)  >  0 if *D*_*S*_  >  (20 + 15*d*)/6≈3.33 + 2.5*d*. For this example, the regions of optimality of the various hiring options from Proposition 2 are depicted in Figure 1.

## 5 SEQUENTIAL EFFORTS

Up to now, we only considered agents that deliver their efforts simultaneously. Alternatively, a firm can induce independent agents to work sequentially. For two agents, Section 3 shows the solution to the principal's problem for simultaneous effort. If the principal employs agents in series, the principal will employ a second agent (i.e., Agent 2) only if the first agent (Agent 1) failed because in our setting, it is sufficient that one agent succeeds. Hence, the principal can benefit from the real option of not employing the second agent after observing the first agent's success. Yet sequential efforts come at a cost for the principal reflected by the discounting of the project's value realized at a later point in time. Specifically, P's expected profit of employing agents sequentially is

(5) $$\max\{0,p_{1}(D_{S}-w_{S}^{1})-d\}+(1-p_{1})\delta \;\underset{=:\Pi_{2}}{\underbrace{\max\{0,p_{2}(D_{S}-w_{S}^{2})-d\}}},$$

where *p*_1_  ∈  {*p*_*L*_,*p*_*H*_} and *p*_2_  ∈  {*p*_*L*_,*p*_*H*_} depict the probability of success of Agents 1 and 2, respectively, and *δ*  ∈  [0,1] is the discount factor.

In order to incentivize Agent 2 to exert high effort, after observing the first agent's success, the principal sets $w_{S}^{2}=W$, and $w_{S}^{2}=0$, to induce low effort. Solving by backward induction (at the end of the first period, the second period becomes the current period, so no discounting is needed here), the principal generally employs the second agent if and only if

(6) $$d<\max\{p_{L}D_{S},p_{H}(D_{S}-W)\}=:d^{0},$$

and the principal will induce high effort by Agent 2 if

(7) $$D_{S}>\frac{p_{H}W}{p_{H}-p_{L}}.$$

Let $\Pi_{2}^{*}\geq 0$ denote the maximized expected profit generated by the second agent. In particular, $\Pi_{2}^{*}>0$ if *d*  <  *d* ^0^. For *d*  ≥  *d* ^0^, P does not hire a second agent, so $\Pi_{2}^{*}=0$. Consequently, for *d*  ≥  *d* ^0^, the second agent is irrelevant and P's decision whether to hire the first agent is also determined by condition (6). If this condition holds, P hires two agents sequentially, if not, no agent is hired. Hence, sequential employment of two agents always dominates committing to employ a single agent.

To motivate high effort by the first agent the principal sets $w_{S}^{1}=W$, and $w_{S}^{1}=0$ else. Inducing high effort by Agent 1 is optimal if

(8) $$\Pi_{2}^{*}<\frac{1}{\delta }\left(D_{S}-\frac{p_{H}W}{p_{H}-p_{L}}\right).$$

The discount factor critically affects whether the principal prefers simultaneous or sequential efforts, and sequential efforts become more favorable for increasing values of *δ*. Consider the case when the principal prefers two high‐effort agents under both regimes and let Π(*H*,*H*)^seq^ and Π(*H*,*H*)^sim^ denote the principal's expected profits of agents working sequentially, or simultaneously, respectively. Comparing the expected profits yields that the decision whether high‐effort agents should work simultaneously or in series always depends on the discount factor, as captured in the following Proposition 3. Similar comparisons can be made for the whole parameter region, with agents not necessarily providing high effort.

Proposition 3 For high‐effort agents, the discount factor that determines whether the principal prefers agents to work simultaneously or sequentially has always an interior solution. Particularly,

(9) $$\Pi {\left(H,H\right)}^{\text{seq}}>\Pi {\left(H,H\right)}^{\text{sim}}\;\Leftrightarrow \;\delta >\frac{p_{H}[(1-p_{H})D_{S}-W]-d}{\left(1-p_{H})(p_{H}D_{S}-p_{H}W-d\right)}=:\bar{\delta },$$

where $0<\bar{\delta }<1$.

**Figure 2:**
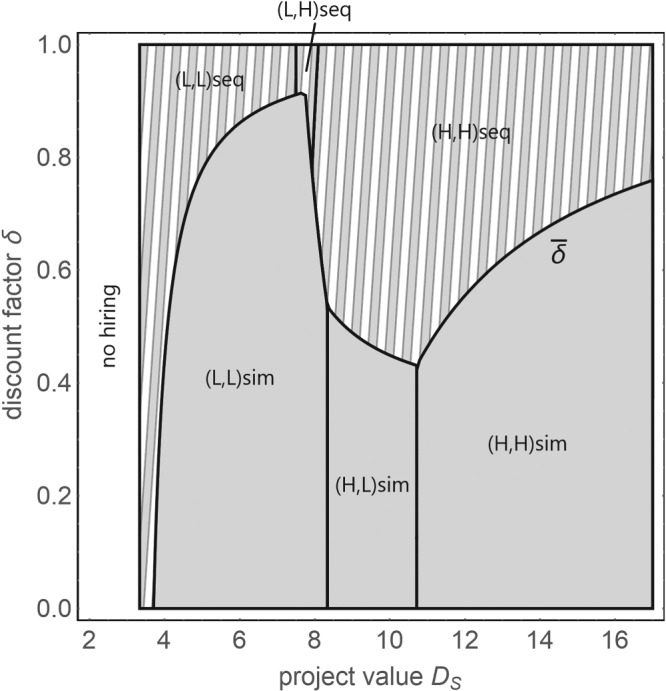
Optimal strategy with two agents for varying magnitudes of the project's value D_S_ and the discount factor δ. The parameters are p_L_  =  1/10, p_H_  =  3/10, and d  =  1/3

Figure 2 presents an example of the principal's preferred strategy for two agents for varying magnitudes of the project's value and the discount factor.

## 6 ASYMMETRIC AGENTS

In our analysis above, all agents are homogeneous. In this section, we allow agents to differ in their ability. More specifically, let us denote a “good' agent by *G* and a “bad” agent by *B*. When P hires agents, she does not know the agents' type. However, the fraction of good agents, *q*, is public knowledge.

Both types of agents can either exert high (*H*) or low (*L*) effort, and their effort can result in success or failure. The respective probabilities of success are 0  ≤  *p*_*GL*_  <  *p*_*GH*_  ≤  1 for *G*, and 0  ≤  *p*_*BL*_  <  *p*_*BH*_  ≤  1 for *B*, where *p*_*BL*_  <  *p*_*GL*_ and *p*_*BH*_  <  *p*_*GH*_. Additionally, we assume that

(10) $$p_{GH}-p_{GL}\geq p_{BH}-p_{BL}\;(\text{i.e.,}\;p_{GH}-p_{BH}\geq p_{GL}-p_{BL}),$$

which expresses that *G* benefits more from high effort than *B* 4. Additionally, exerting high effort can be more costly for a bad agent. Although, as in the previous sections, the costs of providing high effort are *c*(*H*)  =  1 for a good agent, *b*  ≥  1 captures the monetized value of effort for a bad‐type agent.^5^ In this section, the agents are risk neutral.

### 6.1 Single agent of unknown type

The incentive constraints for the two types of agents are

(11) $$G:\;p_{GH}w_{S}-1\geq p_{GL}w_{S}\;\Rightarrow \;w_{S}\geq \frac{1}{p_{GH}-p_{GL}}:=M^{G},$$

(12) $$B:\;p_{BH}w_{S}-b\geq p_{BL}w_{S}\;\Rightarrow \;w_{S}\geq \frac{b}{p_{BH}-p_{BL}}:=M^{B},$$

where we already incorporated that w_F_=0 in equilibrium. Assumption (10) and *b*  ≥  1 guarantee that *M* ^*G*^  ≤  *M* ^*B*^. Hence, P finds it easier to motivate *G* to exert high effort. In fact, if *w*_*S*_  ≥  *M* ^*B*^ holds, both types select *H*, but if *M* ^*G*^  ≤  *w*_*S*_  <  *M* ^*B*^, only *G* selects *H* and *B* selects *L*. Alternatively, if rewards are too low, that is, *w*_*S*_  <  *M* ^*G*^, both types select *L*.

Consequently, P has three options:

(i) *w*_*S*_  =  *M* ^*B*^. Then P's expected profit is
  (13) $$\begin{array}{ccc} qp_{GH}(D_{S}-M^{B}) & +(1-q)p_{BH}(D_{S}-M^{B})-d & \\ & =\underset{=:{\bar{p}}_{H}}{\underbrace{\left[qp_{GH}+(1-q)p_{BH}\right]}}(D_{S}-M^{B})-d. \end{array}$$
(ii) *w*_*S*_  =  *M* ^*G*^. Then P's expected profit is
  (14) $$\begin{array}{ccc} qp_{GH}(D_{S}-M^{G}) & +(1-q)p_{BL}(D_{S}-M^{G})-d & \\ & =\underset{=:\bar{p}}{\underbrace{\left[qp_{GH}+(1-q)p_{BL}\right]}}(D_{S}-M^{G})-d. \end{array}$$
(iii) *w*_*S*_  =  0. Then P's expected profit is
  (15) $$\begin{array}{c} qp_{GL}(D_{S}-0)+(1-q)p_{BL}(D_{S}-0)-d\\ =\underset{=:{\bar{p}}_{L}}{\underbrace{\left[qp_{GL}+(1-q)p_{BL}\right]}}D_{S}-d, \end{array}$$
  where ${\bar{p}}_{H}>\bar{p}>{\bar{p}}_{L}$.

Comparing P's expected profits reveals

(16) $$\text{(i)}>\text{(ii)}\;\Leftrightarrow \;D_{S}>\frac{{\bar{p}}_{H}M^{B}-\bar{p}M^{G}}{{\bar{p}}_{H}-\bar{p}}:=D_{1},$$

(17) $$\text{(i)}>\text{(iii)}\;\Leftrightarrow \;D_{S}>\frac{{\bar{p}}_{H}M^{B}}{{\bar{p}}_{H}-{\bar{p}}_{L}}:=D_{2},$$

(18) $$\text{(ii)}>\text{(iii)}\;\Leftrightarrow \;D_{S}>\frac{\bar{p}M^{G}}{\bar{p}-{\bar{p}}_{L}}:=D_{3}.$$

It is easy to show that *D*_1_  ≥  *D*_2_  ≥  *D*_3_ if and only if *b* is sufficiently large, that is,

(19) $$b>\frac{\bar{p}}{{\bar{p}}_{H}}\cdot \frac{\left(p_{BH}-p_{BL}\right)}{\left(p_{GH}-p_{GL}\right)}\cdot \frac{{\bar{p}}_{H}-{\bar{p}}_{L}}{\bar{p}-{\bar{p}}_{L}}.$$

In that case, if *D*_*S*_  ≥  *D*_1_, (i) is best; if *D*_3_  ≤  *D*_*S*_  ≤  *D*_1_, (ii) is best; and if *D*_*S*_  ≤  *D*_3_, (iii) is best. If *b* is sufficiently small so that (19) does not hold, (iii) is best if *D*_*S*_  <  *D*_2_ and (i) is best if *D*_*S*_  >  *D*_2_. Condition (19), however, is only informative if the rhs is larger than 1.

### 6.2 Two agents of unknown type

When P employs two agents, she has the same three options of setting the prizes:

(i) *w*_*S*_  =  *M* ^*B*^, so that both types select *H* and P's expected profit is
  (20) $$\tilde{\pi }(H,H)={\bar{p}}_{H}\left\{D_{S}(2-{\bar{p}}_{H})-2M^{B}\right\}-2d,$$
  where $\tilde{\pi }(a_{G},a_{B})$ denotes P's expected profit with two agents if any G‐type agent provides *a*_*G*_ and any B‐type agent provides *a*_*B*_, with *a*_*G*_, *a*_*B*_  ∈  {*L*,*H*}.
(ii) *w*_*S*_  =  *M* ^*G*^, so that *G* selects *H* and *B* selects *L*. Then P's expected profit is
  (21) $$\tilde{\pi }(H,L)=\bar{p}\left\{D_{S}(2-\bar{p})-2M^{G}\right\}-2d.$$
(iii) *w*_*S*_  =  0, so that both types select *L*.
  (22) $$\tilde{\pi }(L,L)={\bar{p}}_{L}D_{S}(2-{\bar{p}}_{L})-2d.$$

Comparing expected profits of the three options, we find

(23) $$\tilde{\pi }(H,L)>\tilde{\pi }(L,L)\;\Leftrightarrow \;D_{S}>\frac{2\bar{p}M^{G}}{\bar{p}(2-\bar{p})-{\bar{p}}_{L}(2-{\bar{p}}_{L})}:={\tilde{D}}_{3},$$

(24) $$\tilde{\pi }(H,H)>\tilde{\pi }(L,L)\;\Leftrightarrow \;D_{S}>\frac{2{\bar{p}}_{H}M^{B}}{{\bar{p}}_{H}(2-{\bar{p}}_{H})-{\bar{p}}_{L}(2-{\bar{p}}_{L})}:={\tilde{D}}_{2},$$

(25) $$\tilde{\pi }(H,H)>\tilde{\pi }(H,L)\;\Leftrightarrow \;D_{S}>\frac{2{\bar{p}}_{H}M^{B}-2\bar{p}M^{G}}{{\bar{p}}_{H}(2-{\bar{p}}_{H})-\bar{p}(2-\bar{p})}:={\tilde{D}}_{1},$$

where

(26) $${\tilde{D}}_{1}>{\tilde{D}}_{2}>{\tilde{D}}_{3}\;\text{if}\;b>\frac{\bar{p}}{{\bar{p}}_{H}}\frac{\left(p_{BH}-p_{BL}\right)}{\left(p_{GH}-p_{GL}\right)}\frac{\left[{\bar{p}}_{H}(2-{\bar{p}}_{H})-{\bar{p}}_{L}(2-{\bar{p}}_{L})\right]}{\left[\bar{p}(2-\bar{p})-{\bar{p}}_{L}(2-{\bar{p}}_{L})\right]}=:\tilde{b}.$$

If $\tilde{b}\leq 1$, (26) holds, as *b*  >  1 per assumption. If *D*_*S*_  =  0, we find $\tilde{\pi }(L,L)>\tilde{\pi }(H,L)>\tilde{\pi }(H,H)$ because *M* ^*B*^  ≥  *M* ^*G*^ and ${\bar{p}}_{H}>\bar{p}$. However, all three expected profits are linearly increasing in *D*_*S*_, where $\partial \tilde{\pi }(H,H)/\partial D_{S}>\partial \tilde{\pi }(H,L)/\partial D_{S}>\partial \tilde{\pi }(L,L)/\partial D_{S}$ for all *D*_*S*_  >  0. Consequently, if *D*_*S*_ is sufficiently high, $\tilde{\pi }(H,H)$ is best for P.

More specifically, if the bad agent's costs of high effort are moderate, that is, $1\leq b\leq \tilde{b}$, P will want both agents to exert the same level of effort, because for $D_{S}\in [0,{\tilde{D}}_{2}]$, $\tilde{\pi }(L,L)$ is best, and $\tilde{\pi }(H,H)$ is best for $D_{S}\geq {\tilde{D}}_{2}$. Else, if *b* is sufficiently high, that is, $b>\tilde{b}$, there exists a region where asymmetric contracts are preferred by P, in the sense that a G‐type agent exerts high effort, whereas a B‐type agent exerts low effort. That is, for $b>\tilde{b}$, $\tilde{\pi }(L,L)$ is best for $D_{S}\in [0,{\tilde{D}}_{3}]$, $\tilde{\pi }(H,L)$ for $D_{S}\in [{\tilde{D}}_{3},{\tilde{D}}_{1}]$, and $\tilde{\pi }(H,H)$ for $D_{S}\geq {\tilde{D}}_{1}$.

As a consequence of the result above, we can conclude that for increasing *b*, which can be interpreted as an indicator for the agents' heterogeneity, asymmetric contracts are more likely to appear. Formally, if $b>\tilde{b}$, ${\tilde{D}}_{1}$ is increasing in *b*, so the interval for *D*_*S*_, where $\tilde{\pi }(H,L)$ is best, becomes larger. Intuitively, as it becomes more expensive to motivate a B‐type agent to exert high effort, the (*H*,*H*) equilibrium becomes less attractive.

## 7 SUPPORT BY THE PRINCIPAL

### 7.1 Support with two homogeneous agents

In the basic model, the agents' effort choice determines the probability of success. However, in many situations, P has an opportunity to support agents in order to be successful, for example, by providing guidance, expertise, information, or better working conditions. For example, a university provides its faculty members with an office, furniture, equipment, and other help in research. To induce a researcher to publish more and more, however, increasing increments of support will be needed. Because P cannot observe whether agents provided high or low effort, support will influence the probability of success associated with both types of effort. We first model support in a simple manner, such that both probabilities are multiplied by a factor denoted by *z*. Consequently, an agent exerting high effort will be successful with probability *z*·*p*_*H*_, and the low‐effort agent with probability *z*·*p*_*L*_. If P selects *z*  =  1, the agents receive no support from the principal. More specifically, we extend the basic model so that P can influence the probability of success by a factor $z\in [1,\bar{z}]$ with $1<\bar{z}<1/p_{H}$. Providing support will usually induce costs for P. Let *C*(*z*) denote direct costs P has to bear for maintaining support at level *z*, where *C*(1)  =  0 and *C*(*z*) is increasing and convex in *z* (see also Laux, 2017). In this section, we will restrict our attention to equilibria in pure strategies with symmetric contracts. Before the agents choose their effort, they are assumed to know the level of support P will provide.

The PCs for agents supplying high or low effort are 

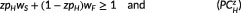


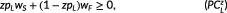
 respectively.

If two agents exert low effort, P does not have to compensate the agents, that is, *w*_*F*_  =  *w*_*S*_  =  0, and P's expected profit is $\pi (L,L)=(2zp_{L}-z^{2}p_{L}^{2})D_{S}-C(z)-2d$. Thus, the optimal level of support *z* ^∗^ will solve the FOC

(27) $$2p_{L}(1-z^{*}p_{L})D_{S}=C^{\prime }(z^{*}).$$

At the optimum, the marginal increase in the expected profit by providing support equals the marginal cost of support.

If P wants the agents to exert high effort, P's optimization problem is subject to the additional incentive constraint, 

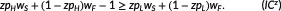



If (*IC* ^*z*^) holds, this implies that (*PC* _*H*_ ^*z*^) holds. As P will set *w*_*F*_  =  0 at the optimum, the optimization can be stated as

(28) $$\max_{w_{S},z}\;(2zp_{H}-z^{2}p_{H}^{2})D_{S}-2zp_{H}w_{S}-C(z)$$

(29) $$\text{s.t.}\;(IC^{z})\;\Leftrightarrow \;zp_{H}w_{S}-1\geq zp_{L}w_{S}\;\Leftrightarrow \;w_{S}\geq \frac{W}{z}.$$

P will optimally set *w*_*S*_ to its lower bound, *w*_*S*_  =  *W*/*z*. The first derivative of (28) with respect to *z* then yields

(30) $$2p_{H}(1-zp_{H})D_{S}-2p_{H}w_{S}-2zp_{H}\frac{\partial w_{S}}{\partial z}-C^{\prime }(z).$$

Apparently, increasing support affects P in various ways. The first term accounts for the higher probability of success and, thus, higher expected profits. The second term, however, indicates that support also increases the probability that P has to pay compensation, which reduces her expected profits. On the other hand, the prize P has to award for success, *w*_*S*_  =  *W*/*z*, which becomes smaller as support increases, so that the partial derivative is negative and thus the third term positive. Finally, the last term depicts the marginal cost of support. Substituting the optimal prize, we find that the second and third terms cancel each other out, and so, the FOC with respect to support simplifies to

(31) $$2p_{H}(1-zp_{H})D_{S}=C^{\prime }(z).$$

Again, the marginal increase in expected profits equals the marginal cost of providing support.

If *both* As had to be successful in order to constitute a project's success, the likelihood ratio would be

(32) $$\frac{Pr(\text{both agents succeed}|(L,L))}{Pr(\text{both agents succeed}|(H,H))}=\frac{{\left(zp_{L}\right)}^{2}}{{\left(zp_{H}\right)}^{2}}=\frac{p_{L}^{2}}{p_{H}^{2}},$$

which is independent of *z*. However, in our setting,

(33) $$\begin{array}{ccc} \frac{Pr(\text{at least one agent succeeds}|(L,L))}{Pr(\text{at least one agent succeeds}|(H,H))} & =\frac{1-{\left(1-zp_{L}\right)}^{2}}{1-{\left(1-zp_{H}\right)}^{2}} & \\ & =\frac{p_{L}(2-zp_{L})}{p_{H}(2-zp_{H})}, \end{array}$$

which does depend on *z*.

### 7.2 Diminishing returns of support

We now model P's influence on the probabilities of success via a concave power function. P can choose support *u*  ∈  [0,1] such that an agent exerting high effort will be successful with probability $1+p_{H}-p_{H}^{u}$. Low values of *u* therefore indicate a low level of support, whereas high levels of *u* indicate high support. For instance, if P selects *u*  =  0, the probability of success for an agent exerting high effort remains *p*_*H*_; that means that the agent does not receive any “help” from P. In contrast, *u*  =  1 indicates full support. In that case, an agent exerting high effort will certainly be successful.

Because P cannot observe whether agents provided high or low effort, support will influence both types of agents. More precisely, when P chooses support *u*, a low‐effort agent will achieve success with probability $k(1+{\hat{p}}_{L}-{\hat{p}}_{L}^{u})$ with *k*  <  1, where in case of no support, that is, *u*  =  0, $k{\hat{p}}_{L}=p_{L}$. Even if P decides to fully support the agents, so *u*  =  1, an agent exerting low effort will not always be successful because *k*·1  <  1. Also, $k{\hat{p}}_{L}<p_{H}$ for all *k*  <  1, and thus, $k{\hat{p}}_{L}^{u}<p_{H}^{u}$ for all *k*  <  1 and all 0  ≤  *u*  ≤  1. Again, in our setting, support influences the likelihood ratio of success, which, in consequence, leads to different incentives as compared with the basic model in Section 2. We will later refrain from assuming costly support, that is, we shall assume *C*(*u*)  =  0∀ *u*, in order to highlight potential negative consequences of supporting that can arise even without any direct costs.

The participation constraints for a single agent supplying high or low effort are 

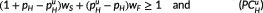


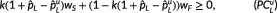
 respectively. If P cannot observe the agent's action and wants the agent to exert high effort, P's optimization problem is subject to the additional incentive constraint, 

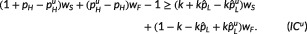



If (*IC* ^*u*^) holds, this implies that (*PC* _*H*_ ^*u*^) holds. So, if P wants the agent to exert high effort, she solves the optimization problem

(34) $$\max_{w_{S},w_{F},u}\left\{\left(1+p_{H}-p_{H}^{u})(D_{S}-w_{S})-(p_{H}^{u}-p_{H})w_{F}-C(u\right)\right\}$$

(35) $$\begin{array}{ll} \text{s.t.}\; & (1+p_{H}-p_{H}^{u}-k-k{\hat{p}}_{L}+k{\hat{p}}_{L}^{u})w_{S}\\ & \geq (1+p_{H}-p_{H}^{u}-k-k{\hat{p}}_{L}+k{\hat{p}}_{L}^{u})w_{F}+1. \end{array}$$

Because $(1+p_{H}-p_{H}^{u}-k-k{\hat{p}}_{L}+k{\hat{p}}_{L}^{u})>0$, we can infer that P will optimally set *w*_*F*_  =  0. Consequently, the optimization problem can be rewritten as

(36) $$\max_{w_{S},u}\left\{\left(1+p_{H}-p_{H}^{u})(D_{S}-w_{S})-C(u\right)\right\}$$

(37) $$\begin{array}{ll} \text{s.t.}\; & (1+p_{H}-p_{H}^{u}-k-k{\hat{p}}_{L}+k{\hat{p}}_{L}^{u})w_{S}\geq 1\;\Leftrightarrow \\ & w_{S}\geq \frac{1}{1+p_{H}-p_{H}^{u}-k-k{\hat{p}}_{L}+k{\hat{p}}_{L}^{u}}. \end{array}$$

P will optimally set *w*_*S*_ to its minimum, $w_{S}=1/(1+p_{H}-p_{H}^{u}-k-k{\hat{p}}_{L}+k{\hat{p}}_{L}^{u})$.

In order to highlight negative consequences of support, we now assume that support is free, *C*(*u*)  =  0 ∀*u*. Substituting the prizes into (36), the problem becomes

(38) $$\max_{u}\left\{(1+p_{H}-p_{H}^{u})\left(D_{S}-\frac{1}{Q(u)}\right)\right\},$$

where $Q(u):=1+p_{H}-p_{H}^{u}-\left(k+k{\hat{p}}_{L}-k{\hat{p}}_{L}^{u}\right)>0$. Thus, the derivative with respect to *u* is

(39) $$-\;p_{H}^{u}\ln(p_{H})\left(D_{S}-\frac{1}{Q(u)}\right)+\frac{\left(1+p_{H}-p_{H}^{u})(-p_{H}^{u}\ln(p_{H})+k{\hat{p}}_{L}^{u}\ln({\hat{p}}_{L})\right)}{Q{\left(u\right)}^{2}}.$$

So, for the derivative to be positive, we need that

(40) $$\begin{array}{ccc} & (1+p_{H}-p_{H}^{u})\left[k{\hat{p}}_{L}^{u}\ln({\hat{p}}_{L})-p_{H}^{u}\ln(p_{H})D_{S}\left(1+p_{H}-p_{H}^{u}-2(k+k{\hat{p}}_{L}+k{\hat{p}}_{L}^{u})\right)\right] & \\ & \;-p_{H}^{u}\ln(p_{H})(k+k{\hat{p}}_{L}-k{\hat{p}}_{L}^{u})\left(D_{S}(k+k{\hat{p}}_{L}-k{\hat{p}}_{L}^{u})+1\right)>0. \end{array}$$

Condition (40) holds for *k* sufficiently small or *D*_*S*_ sufficiently large. Only then will P optimally set *u*  =  1, that is, P will choose full support. However, if, for instance, *D*_*S*_ is sufficiently small, P will optimally refrain from providing support. From the basic model in Section 2.1, we know that if P wants the single agent to exert low effort, she will set *w*_*F*_  =  *w*_*S*_  =  0. In that case, maximum support is optimal, so that *u*  =  1 and P's expected profit is $k(1+{\hat{p}}_{L}-{\hat{p}}_{L}^{1})D_{S}=kD_{S}$.

## 8 CONCLUDING REMARKS

We analyze a multiagent model where it only matters to P whether at least one A was successful. If P can also decide on the size of the workforce, she faces a trade‐off employing an additional agent. Every additional agent increases the probability that at least one agent is successful. However, besides additional administration costs P has to bear, as the number of agents increases, the number of potential rewards P has to pay increases too. We identify conditions when P refrains from employing additional agents but would rather employ a single agent that exerts high effort. Also, we show that the number of agents preferably employed by P is not necessarily monotone in the project's value for the principal. Usually, higher project values increase the principal's demand for additional agents. However, depending on the administration costs, it is possible that when the principal's value from the project increases, she prefers a single (high‐effort) agent over two (low‐effort) agents. As an extension, we then consider sequential efforts by agents. We further allow for asymmetric agents, where P ex ante does not know the agents' abilities. We then allow P to support (help) the agents by increasing their success probability. We consider support that multiplies the probabilities, as well as one that exponentializes them.

We believe that our analysis provides various avenues for further extensions and future research. In one such scenario, P may know the agents' types, reflected in their probabilities of success and/or their costs of exerting high effort, but be required (by law) to offer all an identical contract.

Although we assumed that the agents work on the same task/project independently, one can conceive of situations where the agents' achievements are positively correlated (either due to common external factors or due to some degree of team work). In contrast to our setting, a principal can then benefit from relative performance evaluation. There are also scenarios where, instead of asking all agents to try to (successfully) complete the entire task, the principal splits it and gives each agent a different subtask. That will require all agents to be successful, but if so, the project can be completed faster. This is the motivation behind outsourcing, subcontracting, division of labor, and specialization.

## ENDNOTES

1 The principal's main goal in Laux (2017) is to use support to supplement monitoring to make inferences about the agents' abilities so as to decide who to retain employed.

2 If, in contrast, *p*_*H*_  <  *p*_*L*_(2 − *p*_*L*_), the joint probability of success of two low‐effort agents is larger than the probability of success of one high‐effort agent. Because P does not need to compensate agents providing low effort, incentivizing one agent to work hard is always a dominated strategy.

3 That is, $V_{1}=\min\{A_{1},A_{2},V_{1},V_{2},V_{3},V_{4},V_{5}\}$.

4 Presumably, relative to the probabilities of success with symmetric agents, *p*_*BH*_  ≤  *p*_*H*_  ≤  *p*_*GH*_ and *p*_*BL*_  ≤  *p*_*L*_  ≤  *p*_*GL*_.

5 Hence, if *p*_*GH*_  =  *p*_*H*_, all agents in earlier sections were *G*‐type agents.

## APPENDIX 1

### 1.1

Proof of Lemma 1 The rhs of (3) is increasing in the number of agents if

(A1) $$\begin{array}{ccc} {\left(\frac{1-p_{L}}{1-p_{H}}\right)}^{n} & \geq \frac{1+np_{H}}{1+np_{L}}\;\Leftrightarrow \;\underset{=:f(p_{L},n)}{\underbrace{{\left(1-p_{L}\right)}^{n}(1+np_{L})}} & \\ & \geq \underset{=:f(p_{H},n)}{\underbrace{{\left(1-p_{H}\right)}^{n}(1+np_{H})}}. \end{array}$$

Calculating the first derivative of *f*(*p*,*n*) with respect to its first argument yields

(A2) $$\frac{\partial f(p,n)}{\partial p}=-\;n(1+n){\left(1-p\right)}^{n-1}p.$$

Thus, for *p*  ∈  (0,1) and *n*  ≥  1, this derivative will always be negative, and *f*(*p*,*n*) is decreasing in *p*. Because 0  <  *p*_*L*_  <  *p*_*H*_  <  1, that means that *f*(*p*_*L*_,*n*)  >  *f*(*p*_*H*_,*n*) for all *n*  ≥  1, and consequently, the rhs of (3) is increasing in the number of agents employed. □

Proof of Proposition 1 P's expected profit in case of asymmetric contracts for two symmetric agents is

(A3) $$\pi (H,L)=p_{H}\left[D_{S}-W\right]+(1-p_{H})p_{L}D_{S}-2d,$$

(A4) $$=(p_{H}+p_{L}-p_{H}p_{L})D_{S}-p_{H}W-2d,$$

where

(A5) $$\pi (H,L)>\pi (L,L)\;\Leftrightarrow \;D_{S}>\frac{p_{H}}{{\left(p_{H}-p_{L}\right)}^{2}(1-p_{L})}:=A_{1}\;\text{and}$$

(A6) $$\pi (H,H)>\pi (H,L)\;\Leftrightarrow \;D_{S}>\frac{p_{H}}{{\left(p_{H}-p_{L}\right)}^{2}(1-p_{H})}:=A_{2}.$$

For 0  <  *p*_*L*_  <  *p*_*H*_  <  1, there exists a nonempty region, that is, *D*_*S*_  ∈  [*A*_1_,*A*_2_], with *A*_1_  <  *D*_*S*_(2)  <  *A*_2_, where P prefers asymmetric contracts for two symmetric agents. □

Proof of Proposition 2 Part (i) in text. The proofs for the other parts work analogously. For (iv), the threshold that *π*(*H*) is positive is *D*_*S*_  >  *W* + *d*/*p*_*H*_. However, for *d*  >  *d*_3_, we find *D*_*S*_(1)  <  *W* + *d*/*p*_*H*_. Hence, both a low‐effort and a high‐effort single agent yield negative profits for *D*_*S*_  <  *D*_*S*_(1).

Proof of Proposition 3 Inspecting the threshold in (9), on the one hand, $\bar{\delta }<1$ if *p*_*H*_(*D*_*S*_ − *W*)  >  *d*, which must hold because else hiring a high‐effort agent would result in negative expected profits. Hence, the denominator in (9) is positive, and $\bar{\delta }>0$ if *D*_*S*_  >  (*p*_*H*_ *W* + *d*)/[*p*_*H*_(1 − *p*_*H*_)]. This condition is necessary for P ex ante to prefer high effort by two simultaneous agents over a single high‐effort agent. Because we only consider scenarios where (*H*,*H*)^sim^ is P's preferred strategy in the simultaneous efforts setting, we conclude that $0<\bar{\delta }<1$. □
